# Division of the Teeth into Two Classes. One Called the Temporary or Milk; the Other the Adult or Permanent. Period of the Change of the Teeth Called Molting. Error of Those Who Believe in a Third Dentition

**Published:** 1845-06

**Authors:** 


### ARTICLE IV.

*Division of the Teeth into Two Classes. One called the Temporary or Milk; the other the Adult or Permanent. Period of the Change of the Teeth called Molting. Error of those who believe in a Third Dentition.* Translated from the French of Delabarre, by * A.*

Should we be astonished that the ancients invented and repeated such absurd stories about dentition; when we reflect, that, even in these latter days, authors on physiology have not given themselves the trouble to notice those physiological differences, by which we distinguish the teeth appertaining to one dentition from those belonging to the other? Prejudicial errors are, therefore, inevitable, if we neglect to study the proper characteristics, not only of each class of teeth, but also each individual, in the series of which that class is composed. A knowledge of the irregularities of *Odontocie* is not the less indispensable. The same may be said of the number of the teeth, for Bichat* from having followed *Bertin*, has falsely asserted, that the first temporary molar is replaced by the two bicuspides. *M. Baums* is likewise deceived.

I will, therefore, attempt to fill up the blanks, which, in most of our elementary books, exist on this subject; thus, each jaw of the infant is provided, until about five and a half years, with ten teeth, which are called milk teeth; but which are better named temporary or deciduous teeth, since they are soon re-

* Bichat (Anat Genival, page 94 a' 97) says, that there are twenty-four teeth belonging to first dentition, and, consequently, he comprises in this the first permanent molares, that appertain to the second, and do not shoot up till about five and a half years. In admitting three deciduous molares for each place, he admits twelve temporary teeth for each jaw, while a little further on, he acknowledges that there are only eight which are to be replaced. Had he consulted the works of dentistry, he would have avoided this mistake.

I have, three or four times, seen a supernumerary tooth of first dentition, but this is a circumstance of rare occurrence. I have also seen several instances of children in which one or two temporary incisions were wanting.

placed by others. This change is designated by the word molting or shedding.

The color of the primitive is generally inclined to a light blue. The volume of the six anterior is not ordinarily less than the space occupied by the same number of the smallest of the adult teeth. Their cutting edges are finer, their crowns sounder and better set. The four milk molares do not have the same forms as the four bicuspides, by which they are to be replaced; they rather resemble the adult molares, but their surfaces do not present the same appearance. The first of them has three points on the side next the tongue, and two on the side next the cheek. This tooth appears to be compressed from without and within. The second is rounder and exposes five points, three on the outer, and two on the inner side. The faces of these two teeth, which correspond to the cheek, are flattened and inclined in a remarkable manner; near the neck we perceive a sort of gibbosity.

These teeth in the inferior jaw have two roots, which are very strong and much separated from each other; in the superior jaw they have three. The first of these molares, I mean the one nearest the conoid, being about a third less than that which followed it, has led some authors to admit a small and a great molar of first dentition; a just distinction, but one that has caused those who have not rightly understood it, to make many mistakes. These teeth as well as the conoids are liable not to be renewed at the usual time of molting, and, in this case they frequently endure for many years.

L'Ecluse (page 114) pretends, that the milk-teeth are insensible, and that if the child experiences any pain in them, it has its seat not in the ganglion, but in the periosteum, which, from some cause has become inflamed. A modern author, of our day, is equally certain, that the temporary teeth, at a certain epoch, cease to be under the nervous influence, *by reason of the obstruction of the nervous fillets that are carried to them.* I have often endeavored to ascertain, if these suppositions had any foundation, but in all the researches that I have made on this subject, both alone and assisted by intelligent pupils, I have always, until the period of molting, traced the nerves which are carried to the temporary class of teeth.

Though anatomical observations had satisfied me of the fact, I still was anxious, that all the young men, who were pursuing my course, should likewise be convinced; in consequence, I made, at two different times and in the presence of those who were found in my visit at Ophelius, the two following decisive experiments that may easily be repeated.

First, I forced a very fine needle into the two central canals of several temporary teeth attacked by caries, and immediately the pain experienced by the child determined me to extract them.

In divers others of six or seven years, with sharp forceps I cut some of the incisors in such a way as to uncover the *ganglion;* I then either had the mouth washed with cold water or irritated the nerve with a needle, or applied a small quantity of nitrate of silver, and, by any of which means, I occasioned much pain. Hence the teeth are sensible, and hence they continue to receive the nerves until the period of moulting.

Still we occasionally meet with temporary teeth whose nerves and vessels have been obstructed, and sometimes even absorbed by the membranes of the teeth of replacement, but this is nothing more than accident, and such teeth are of a remarkably bluish color. The adult teeth, or teeth of the adult, constitute second dentition. The ten anteriors of this class form an order of teeth called teeth of replacement, because they, in effect, replace the entire class of temporaries.

The molares of the adult constitute the class of the permanents; these shoot up only once, and do not require the moulting of any teeth, in order to be replaced in the dental arch, and if one of them is extracted, it is not usually replaced by another; I say, usually, for it sometimes happens that a supernumerary germ, found between its roots, gives birth to another tooth that will afterwards appear.

Each class is composed of several series in which we rank the teeth of the same kind. They take their names either from their use or from their forms. Thus the four anterior teeth are called *incisors* or cuniform, because they are shaped somewhat like wedges. In the superior jaw, we divide them into *great* or *central*, and into *medial* or *lateral*.

In the inferior jaw these teeth are called small, but through a remarkable singularity, the laterals are the largest, even in brutes. Those which are placed after the cuniform, have received a multitude of names, the most common of which are the angular, because they are found in the neighborhood of the angle of the lips; the eye, because they are placed under the angle of the eye, the *canine*, because they are compared to the teeth of dogs; the lacerators, because in carniverous animals they serve to tear in pieces their prey:—finally, we also call them the *cuspidati* or *conoids*, denominations that appear to me to be the best. These teeth are more convex than the cuniform or incisors. Then come the small or semi-molares, better denominated the bicuspides, from their being armed with two points; they therefore differ much from the milk molares, which they have replaced. Finally, we call those which follow, molares or grinders. These have also received the name of *contri-cuspides*, on account of the four eminences that are observed on their crowns. In general, this series of teeth present quadrilateral surfaces, with rounded crowns, their labial faces, and those which correspond to the cheeks, are also round.

The course pursued by nature in first *odontocie*, is not always the same that is followed by her in the second; thus, in one the bicuspides spring up before the conoids or canine, while in the other they do not appear until after these. First dentition, also may terminate in good time, and the second be tardy, so *vice versa.* They are therefore absolutely independent of each other. Hence, children that have had bad *temporary* teeth, may have very excellent adult ones. We may also observe that the primitive teeth are very rarely badly arranged, while those of replacement are very frequently illy adjusted. From the knowledge of the developement of the teeth by means of germs, we perceive, that it is not because a temporary tooth has been removed, that one of the adult teeth come up; but because the absorption and natural moulting of the first only happens, in as much as *the second* is developed; so that were not this to occur, the *primitive* might remain in its place throughout the whole life, even as we sometimes observe is the fact with persons of a very advanced age. But as the leaves that cling to the tree, in autumn, are yellow and withered, so the temporary teeth left in their place, lose, after the third or fourth *lustre*, that whiteness which before constituted their charm; having been found to be destroyed at a certain epoch, nature, after it has passed, appears to grant them with reluctance, a very small quantity of the nutritious juices. We may now easily see that, to the prolonged sojourn and tardy moulting of some of these teeth is to be attributed to the foolishly accredited error of a third dentition. The moulting of the teeth being a law of our organization, the professional man, when called upon to direct the arrangements of the adult teeth, should possess an accurate knowledge of the physiological facts of which I have just given an outline. He ought to know how to act opportunely; but he more frequently ought to remain a positive spectator, in order not to oppose nature by painful and useless operations.

There are certain periods of life destined for the operation of certain phenomena. Thus, respiration is established at birth, the temporary teeth sprout up during the first two years; then they are shed in the course of the second or third lustres; menstruation frequently does not commence until dentition terminates, &c.

It is the first permanent molares that give the signal of *moulting;* they commonly shoot up at from five to six years, (rarely at a later period,) and are placed behind the temporary teeth. It is thus that the all-provident Creator gives us new teeth before we are deprived of those of our childhood.

But the moulting of some of the temporaries may happen very much before the shooting up of the teeth of replacement; this proceeds from the occasionally too precarious developement of the absorbents that enter into the composition of the *dental* matrix; it is, however, only a peculiarity, for, in the majority of subjects, the moulting of a temporary is closely followed by the odontocie of that which should replace it. And, though the shooting up of each series of teeth is susceptible of much variety, still we may distinguish those natural orders in the *odontocie* of replacement, and sometimes the period at which it commences.

First order; sometimes at the age of five years, but more commonly at from six to eight, or even nine, according to the strength of the child, two temporary incisors are moulted, first on the lower jaw, to be replaced by two new teeth. The upper centrals drop out, and their places are soon occupied by two new ones. Then follow the inferior lateral incisors, and in time these are followed by the laterals of the upper jaw, but they do not always alternate thus.

After the eruption of these teeth, there is a repose of a shorter or longer duration, which sometimes continues for two or three years. Then towards the age of nine, ten, twelve, or even fourteen years, the five bicuspides of each jaw drive out the first or small milk molares; then the second bicuspides come up, which determines the moulting of the second temporary molares. Finally, the conoids, or adult canines, seize upon the place of the primitive of the same name. In this order there is a variety, which consists in this, that sometimes the second bicuspides shoot up after the conoids.

*The second natural order* only differs from the first in that the conoids or canine spring up before the first and even the second bicuspides. The first order appears to me to be the most common. The second, next. As to the third, it is altogether less frequent. The second great molares or quadricuspides, usually come up as soon as the conoids are in place, that is to say, about the age of twelve years. But it sometimes happens that they precede them. The eruption of the teeth, moreover, presents irregularities; sometimes we meet with a subject from ten to twelve years, on one of the sides of whose jaws there has been no moulting, while on the other side the twelve have been renewed. The medial line in such a case separates first from second dentition, which are found together at the same time in the same mouth. In some others the teeth have sprung up in a portion of one of the jaws, while in another part there is no indication of them. At the *hospice of Orphelius*, where a great number of children are submitted to my examination, I have seen the bicuspides come up at seven years, and the incisors not break until twelve, &c. &c.

I have generally observed, that both dentitions are more precocious in subjects that are effected with diseases whose seat is in the head, while they are more tardy in those that have mucous or misentene affections.

The set of adult teeth, in a certain number of individuals, is composed of twenty-eight; nevertheless four of these in the majority of cases, only come up at from the twentieth to the twenty-fourth year. But their eruption now and then occurs at a very advanced period of life, for we have had occasion to observe some persons who did not have them until about sixty years, and even at a much older age.

The developement of the teeth has a remarkable influence upon the physiognomy, for it is only as this is gradually affected that the maxillary bones become enlarged, that the angle of the inferior and the tuberosity of the superior jaws are carried more or less behind—and that the perpendicular diameter of the head acquires a greater height.

What a difference between the mouth of a child of five years and that of one of eight or nine; the former is rounded and graceful, the latter stretched and of a much greater size. In effect, the very rapid shooting up of six or eight anterior teeth produces an almost sudden change in physiognomy. The developement of the alveolar edges is effected with so much promptitude, that there no longer appears to be any harmony between the maxillary bones, and those of the rest of the face.

Thus, at the period of second dentition, the sweet expression of the infant is lost, to give place to that of the adult, and the facial angle instead of being open becomes more contracted.

We may remark as a striking change, when a person, after having had all his teeth, finds himself suddenly deprived of them; for the breaches that result, are not only troublesome in the articulation, but also determine a deformation in the traits of the visage. The volume of the teeth belonging to a second dentition, is very variable, and in the human species, is not proportional to the strength of the individual. Thus we sometimes see very robust people with very small teeth, while in others, who are of a weak constitution, these are of an enormous size. The examination of the teeth, with reference to this relation, might give rise to many very interesting physiological considerations, but would lead me too far away from the consideration of the present subject.

We sometimes meet with persons whose palatine arch and inferior jaw are very fully developed, while their teeth are very narrow, and have a very observable space between them, this constitutes a slight irregularity that we should not attempt to remedy.

Although the mouth of man is commonly furnished with twenty-eight or thirty teeth, still it occasionally happens that some of the gums of the adult teeth are either not at all, or very tardily developed. In the latter case the absorbing apparel destroys the temporary tooth above or below it, which has not before been moulted. The conoids, the bicuspides, and the small cuniform are those, as I before observed, which most frequently afford these varieties. I have had occasion to see several aged persons, in whom the teeth spring up at a very late period; especially a lady at seventy years of age, each of whose jaws was endowed with four excellent teeth after the loss of all the others. These were the conoids which had not come up in her youth, and the *dentes sapientiæ* that for a great number of years had remained enclosed in the jaws. Persons little skilled in the art would have taken this for an instance of third dentition.

Had ancient anatomists entertained true notions about the formation of the teeth, and the varieties of nature in the variable epoch of their eruptions, they would not have copied from each other the absurd fables of Eriphius, Euriptoleme, Pyrrhus, &c., each of whom, it is said, had only one tooth occupying the whole of the jaw.*

What shall we say to those, who assure us that each of the daughters of Mithridates had two ranges of teeth, and that this also was the case with *Themachus*? What to those who tell us, that three rows adorned the jaws of *Hercules*, as well as those of the children of the anatomist Columb?

We answer, we would still see these phenomena reproduced, were not dentists careful to act in time, those temporaries whose

* May it not be possible that the text of the ancients has been corrupted by translators? Thus, he calls ***Prussias,*** Monodons, from his having only one tooth in the superior jaw, but he does not say that this tooth occupied the whole of the jaw. We also see, too, many persons to whom no no more remain. As to the rest, the tartar that, from the want of care, collects on the teeth, sometimes unite them in such a way, that a careless observer might easily be led to suppose that they formed one and the same body.

roots were not wholly, or the same as wholly, destroyed, after the eruption of the teeth of replacement, were they not to do this, they would come up sometimes within, sometimes without the arch, which would present an extraordinary number of solid teeth. Latterly, I, with several of my pupils, observed this in a girl of twelve years, from whom I took four temporary incisors, whose roots were still untouched, though the teeth of replacement had made their appearance in the inner side of the circle, and not far off from those that I had extracted. As to the rest, this double or triple range can exist only in the anterior part of the mouth, because it is only here that the teeth are renewed. Ought we not, therefore, to be surprised when we see a modern author of the eighteenth century seriously reproducing these same stories of a *third dentition* at the age of eighty or a hundred years, which are reported by *Mutianus*, Aristotle, Mentzelius, &c.?

We cannot but smile at the simplicity of an ancient author, who says he saw in a child's mouth a tooth of gold, a trick of which there is no need that the physician should be the dupe.

Finally, many persons have reported pretended instances of children or adults, from whom a tooth has been removed, after having been before removed, which was, nevertheless, a second and even a third time replaced.

All these errors have had their source, 1st. In the ignorance of the people; 2d. In the want of skill in physicians, who, even in our day, have paid very little attention to dentition; but it is now time that the falsity of these assertions should be understood, in order that we may not imitate the propagators of these absurd follies, whose credulity has given too many pretexts to the ignorant for extracting crowded teeth, while, at the same time, they have induced their victim to expect that these would be replaced by other and prettier ones; but to the great chagrin of the credulous, and the confusion of the operators, these last are still to come, and in waiting for them, recourse must be had to false ones.

Still nature at one time appears to be avaricious, and at another profuse. There are some individuals who have supernumerary teeth. I saw at the hospice an infant whose first dentition presented six incisors of the inferior jaw; after moulting, these were replaced by four only. In many dissections, I have observed several examples of such irregularities.

Thus, I have met with persons who have had five adult incisors,* three *canine*, or five bicuspides, instead of the ordinary number.

Finally, practice shows us interculary teeth, which are small abortions placed between the central incisors or others, but they really possess the form of the series of teeth in the midst of which they are found. On the 22d of July, 1818, M. Debay, a young surgeon dentist, brought to me a child of nine years, showing a medial incisor between the two superior centrals.

Sometimes there are twin teeth, or teeth with double crowns. I once dissected a very young subject, in which I found a milk incisor presenting this peculiarity.

The roots of some teeth are sometimes so closely entangled, that we have seen two extracted, when it was intended to effect the evulsion of only one. Fix has had engraved quite a number of these sorts of small phenomena.

Finally, we may observe another singularity. We have had at the hospice of orphans a child of eight years old, of an evidently scorbutic temperament, that never, *would any one believe*, had any temporary teeth. This child was carried to the country and died; I was not, therefore, able to ascertain if the germs of the the teeth of replacement existed. Fauchard cites an analogous case. Beaumes, in his excellent treatise on dentition, relates that a beadle of his acquaintance, named Vaizor, (conun de St. Gilles,) never was possessed of a single tooth; Villa likewise reports, that in his own time, Pheneceats never had any teeth.†

* I have just extracted one of these duplicate teeth from a nephew of one of the first *valets de chambre de* S. A. R. MONSIEUR.

† I dare say I shall be accused of obstinate incredulity, but I confess that, I scarcely believe in a *congenital and absolute want* of all the germs of the *temporary and adult*, since I have seen in different hospitals to which I have been attached, many young children that were sick of a cancerous or scorbutic inflammation of the gums, which carried along with it the mortification of a greater or less number of the germs: whence, I conclude that, those in whom we observe a total absence of teeth, may have been deprived of them by similar causes.

These things, however rare, have nothing in them that shocks common sense, but as to fables, let us have poets to please and amuse the idle, among the number of whom the physician should never be found.

The mass of certified facts that I have brought forward, should serve the physician as a basis to establish a certain method of directing the arrangement of the adult teeth. Nature, in effect, directs all her care to the enlargement of the alveolar circle; and in order to effect this end, she combines various means. Let us succinctly recapitulate them: 1st. The general increase of the maxillary bones depending on that of the other bones of the body; 2d. The periosteum of the maxillary bones, very remarkable in the places where these should be especially increased, namely, anteriorly from the medial lines to the first temporary molar, posteriorly from the second molar to the conoid apophysis; 3d. Six small imbricated matrices, each of which contains a tooth steeped in liquid, that, somewhat like a wedge, dilates all the surrounding parts; 4th. *Progressive disimbrication* of these small matrices, as the circle is gradually enlarged; 5th. The developement of an apparel charged to absorb every thing that might oppose *odontocie;* 6th. The eruption of the teeth according to the natural order that I have described, subject however to some varieties.

We shall, in the following article, see what advantage may be derived from the knowledge of these various phenomena.

